# Building community through higher music education: a training program for facilitating musical engagement among older adults

**DOI:** 10.3389/fpsyg.2023.1102446

**Published:** 2023-05-05

**Authors:** Paolo Paolantonio, Stefano Cavalli, Michele Biasutti, Hubert Eiholzer, Aaron Williamon

**Affiliations:** ^1^Department of Research and Development, Conservatory of Southern Switzerland, Lugano, Switzerland; ^2^Centre for Performance Science, Royal College of Music, London, United Kingdom; ^3^Department of Business Economics, Health and Social Care, University of Applied Sciences and Arts of Southern Switzerland, Manno, Switzerland; ^4^FISPPA Department, University of Padua, Padua, Italy; ^5^Faculty of Medicine, Imperial College London, London, United Kingdom

**Keywords:** fourth age, music, health and wellbeing, higher music education, nursing homes

## Abstract

Preparing music students to design and carry out community-based initiatives can be an effective way to enhance their employability and their wellbeing. With a large body of evidence now pointing to the benefits of musical engagement for older adults, both for individuals and for society as a whole, there is considerable opportunity and value in training aspiring professional musicians to work with and on behalf of those in their third and fourth age. This article describes a seminar designed by a Swiss conservatoire in collaboration with local nursing homes involving residents and music university students in a 10-week group music making program. On the basis of the positive results to emerge in terms of health, wellbeing and career preparation, we aim to provide information relevant for colleagues to replicate this seminar in other higher music education institutions. Moreover, this paper aims to shed light on the complexity of designing music students’ training so that they acquire the competences needed to deliver meaningful, community-based initiatives alongside their other professional training commitments, and to provide directions for future research. The development and implementation of these points could foster the increase and sustainability of innovative programs beneficial for older adults, musicians and local communities.

## 1. Background and rationale for the educational innovation

Music conservatoires and universities are tertiary institutions where students are expected to achieve mastery of their instruments and/or specialize in a specific activity such as composition, instrumental/vocal performance or conducting (Perkins et al., 2017). From an international perspective, performance teaching in these institutions is mostly delivered one-to-one, focusing to a significant extent on the acquisition of virtuosic skills (Burwell, 2016; Gaunt, 2011; Haack and Smith, 2000; Perkins et al., 2017). It is widely acknowledged that this educational approach can bring risks in terms of psychological wellbeing (Carey and Grant, 2014; Demirbatir, 2015; Burwell et al., 2017; Bull, 2019) and could be, on its own, inadequate for facilitating a transition into the profession (Smilde, 2018; López-Íñiguez and Bennett, 2020). Consequently, in recent decades efforts have been made worldwide aimed at broadening music students’ curricula and increasing the effectiveness of their learning environments. Considering the European context, in 2001 the Association Européenne des Conservatoires (AEC) began to develop learning outcomes specific to higher music education, and currently three areas of competences are considered: practical, theoretical and generic skills (Association Européenne des Conservatoires, 2017; see also Williamon et al., 2017; Cartwright et al., 2021). At the same time, many authors have suggested that strengthening the presence of music conservatoires and universities in their local communities could be beneficial for students’ employability and wellbeing (Bowles and Jensen, 2018; Gaunt et al., 2021; Perkins et al., 2015; Triantafyllaki and Anagnostopoulou, 2013; Westerlund and Gaunt, 2021).

Preparing musicians of the future to reach out to new audiences and use their performance and teaching competences outside the usual venues (e.g., theaters, concert halls, and music schools) is becoming more and more important for several reasons. The awareness of the potential of arts-based interventions in terms of cultural capital, resilience, equity, health, and wellbeing has grown considerably in recent years, and the literature focusing on music offers much evidence regarding the benefits of engagement with the arts (Fancourt and Finn, 2019). At the same time, the notion of *community music* has become more and more widespread internationally. Based on the premise that “everyone has the right and ability to make and create music” (Veblen and Olsson, 2001, p. 2), it promotes active, inclusive participation in music making, facilitating the engagement of disadvantaged groups and supporting individuals’ identity, belonging, and self-expression.

We consider it important to offer music students competences that can be used to reach out to older adults. Engagement with music, in its different forms, can have positive effects for older people. Regardless of musical background, listening to music can reinforce identity across the transitions of aging (Hays, 2005), providing positive effects on mood, self-esteem, and social relationships (Hays and Minichiello, 2005; Laukka, 2006; MacDonald, 2013). Moreover, attending concerts can provide rewarding experiences, decrease anxiety, and facilitate social interactions (Costa and Ockelford, 2019; Tymoszuk et al., 2020a,b). A wide range of positive effects has been found also in the participation in music making programs. Singing can provide benefits in terms of self-confidence, happiness, and social interactions (Pearce et al., 2016; Lamont et al., 2017; Joseph and Southcott, 2018) and can increase vitality and reduce depression (Varvarigou et al., 2012; Daykin et al., 2018). Making music can have a significant impact on hedonic and eudemonic dimensions of wellbeing (Hallam and Creech, 2016), improving at the same time concentration, memory, attention, and other cognitive abilities (Creech et al., 2013; Seinfeld et al., 2013; Perkins and Williamon, 2014).

However, applying musical skills in community-based settings is not simple, as the benefits perceived from engagement with music derive from complex processes and are significantly influenced by individual needs and specific circumstances (Perkins et al., 2020). Although high standards of musical expertise remain crucial (Costa and Ockelford, 2019; Fancourt and Finn, 2019), it is imperative to provide musicians with a wide range of extra-musical competences and to support them in developing a reflexive attitude, even in terms of professional identity (Renshaw, 2010; Preti and Welch, 2013; Smilde, 2018). In her book *Understanding facilitation: Theory and principles*, Hogan (2002) defined the figure of *facilitator* as a “self-reflective, process-person who has a variety of human, process, technical skills and knowledge” (Hogan, 2002, p. 57). Higgins (2007), discussing the concept of *community music*, argued that “traditional training for musicians was not adequate within the community music arena; competences such as musicianship, workshop-leading skills, project managing, and entrepreneurship were all necessary for the job” (p. 33). Ruud (2012), discussing the potential of community-based musicking practices in terms of disease prevention and health promotion, stressed the need of “a new kind of musician […] a *health musician*, if you will” (p. 95) in possession of adequate skills, competences and values. A contribution focused on older learners suggested “the interpersonal qualities, teaching strategies, skills, and knowledge of leaders and facilitators may be more important, in some cases, than the content itself” (Hallam et al., 2015, p. 20).

Based on the extant research, we ran between 2015 and 2017 *Art for Ages* (A4A), a research program aimed at exploring the experiences of and the perceived effects on residents in nursing homes, as well as the music students involved, of a 10-week group music making program. The findings show that the encounter between these two groups provided mutual benefits. For residents, music plays an important role in their lives (Paolantonio et al., 2021), and the 10-week program elicited positive emotions, provided learning opportunities and facilitated interpersonal relationships (Paolantonio et al., 2020). At the same time, this experience had a positive impact on students’ professional and personal spheres, providing benefits on psychological and physical dimensions as well as offering insights and competences relevant for their musical careers (Paolantonio et al., 2022). Overall, the results of A4A highlight the importance of including opportunities for students to act as music facilitators for older adults in the curricula of music conservatoires and universities.

In this Curriculum, Instruction, and Pedagogy article, we describe the training provided for students enrolled in A4A. We developed a seminar^1^ offered by the Conservatory of Southern Switzerland (*Conservatorio della Svizzera italiana*, Lugano) in the academic years 2015–2016 and 2016–2017. Besides the encouraging results to emerge from A4A, making students aware of the role of engagement with music in the later stages of life and training them accordingly is important for many reasons. Firstly, in many countries populations are aging and life expectancy is very high (World Health Organization, 2018). It is paramount to support active aging, namely “the process of optimizing opportunities for health, participation and security to enhance quality of life as people age” (World Health Organization, 2002, p. 12). As engagement with music can provide significant benefits in terms of health and wellbeing, facilitating access to music for older adults can play an important role. Secondly, providing students with a set of competences to carry out music interventions specific for older adults may significantly expand employment opportunities for musicians. Thirdly, being engaged in community-based activities can be beneficial for students too in terms of identity and interpersonal relationships (Ruud, 2012; Triantafyllaki and Anagnostopoulou, 2013; Perkins and Williamon, 2014; Smilde et al., 2019; Gaunt et al., 2021; Westerlund and Gaunt, 2021; Paolantonio et al., 2022). Finally, through the implementation of this seminar and the development of training activities, we aim to demonstrate how evidence-based courses can be incorporated into curricula in higher music education institutions.

## 2. Pedagogical framework underpinning the educational activity

We designed our seminar considering the concept of situated learning (Lave and Wenger, 1990; Renshaw, 2010; Smilde, 2018) and involving a multi-disciplinary teaching team. We offered music university students, namely people who are expected already to possess a high standard of musical skills, the opportunity to go outside the music conservatory classroom and to work on site, in direct contact with the residents and the nursing homes staff. As we were addressing a frail population (Bortz, 1993; Fried et al., 2001), at least two staff members familiar to the residents were present in each session. This minimized the risk of provoking any harmful experiences to both residents and students. For the same reason, we created a teaching team comprising two lecturers, one in sociology with expertise in aging and one in nursing science. Our teaching team also included a Conservatory of Southern Switzerland teacher with extensive experience in pedagogy and musical ensembles, who acted as workshop leader in each session. Through the activities summarized in Table 1, she introduced students to percussion instruments and repertoires to be included in the music sessions.

**TABLE 1:** The structure of the seminar.

Component	Content
Preliminary training 6 h	Part 1 (2 h) Understanding the aging processes from sociological and medical points of view Part 2 (4 h) Introduction to repertoires and instruments for group music making in nursing homes
Interventions in nursing homes 25 h	10 weekly group music making sessions, with each session structured as follows • Rehearsal (60 min) • Session (60 min) • Debriefing (30 min)

## 3. Learning environments, objectives and pedagogical format

The seminar discussed in this article arises from *Art for Ages* (A4A), a research project led by the Conservatory’s Department of Research and Development in collaboration with the Department of Business Economics, Health and Social Care, University of Applied Sciences and Arts of Southern Switzerland, (Manno, Switzerland) and the Centre for Performance Science, Royal College of Music (London, United Kingdom). The project involved four nursing homes in Southern Switzerland hosting a 10-week program of group music making for their residents. A4A involved Bachelor and Master students from the Conservatory, recruited on a voluntary basis through their enrollment in the elective seminar described here. The research project was conducted between 2015 and 2017, and consequently, the seminar was offered twice: in the academic years 2015–2016 and 2016–2017. It was developed under the framework of establishing collaborations with local nursing homes and providing professional opportunities for students. Each nursing home welcomed our proposal and was supportive. In the following sections, we discuss the training offered to students and describe the musical program carried out in the four homes involved.

### 3.1. Format and contents of the seminar

The seminar was divided into two stages including a preparatory and a practical section, as shown in Table 1. Students attending the preliminary training and taking part in at least eight interventions in nursing homes achieved 2 credits ECTS (European Credit Transfer and Accumulation System) as a part of their 180-credit Bachelor or 120-credit Master degree.

The preliminary training was divided into two parts. The first was led by two lecturers from the Department of Business Economics, Health and Social Care, University of Applied Sciences and Arts of Southern Switzerland (Manno, Switzerland). They have expertise in sociology (Stefano Cavalli) and nursing sciences (Carla Pedrazzani). In a 2-h lecture they conveyed information on aging processes from medical and sociological perspectives, focusing on the heterogeneity of the aged population in terms of socio-economic profiles and physical, psychological and cognitive health (Nelson and Dannefer, 1992; Stone et al., 2017); on the main differences between the “young old”—or third age (Laslett, 1989; Baltes and Smith, 2003)—and the “oldest old”—or fourth age, the “age of frailty” (Lalive d’Epinay and Spini, 2008; Lalive d’Epinay and Cavalli, 2013); on the resources of older adults and the risks of ageism—stereotyping and/or discriminating against an individual or a group on the basis of age (Butler, 1969)—to which they are exposed. The second part involved a Conservatory teacher with extensive experience in pedagogy and musical ensembles (Silvia Klemm), who acted as a workshop leader throughout the 10-week group music making program carried out in each nursing home. In 4 h, she introduced students to the percussion instruments that were to be used by the residents, as well as several songs and exercises to be played or sung. In this way, she created a preliminary repertoire adequate to promote the engagement of residents from the earliest sessions.

The songs and the vocal works were arranged in canon, unison or simple harmonization, depending on the genre and the desires of residents. The percussion instruments were chosen considering their light weight and ease to play. We considered their loudness to avoid disturbing the residents who were not participating in the activities. To involve residents with severe motor impairments, we included several one-handed instruments. We also made an extensive use of drumsticks, which have a considerable potential in musical and motor terms even when used alone. Along with these instruments, everyday objects were used. In this way, residents were offered the opportunity to play “actual” instruments while the availability of familiar objects of modest price helped to create an inclusive and fun atmosphere, encouraging even the shiest residents to take part in making music. Table 2 lists the whole set of percussion instruments included in A4A.

**TABLE 2:** Set of percussion instruments used in *Art for Ages*.

Instruments	Common objects
*Usable with one or two hands* Bells Boomwhackers Cymbals Drumsticks Egg shakers Maracas Rattles Small drums Tambourines	*Usable with one or two hands* Baskets Chairs
*Usable only with two hands* Guiros Triangles	*Usable only with two hands* Graters Spoons

The practical component consisted of taking part in one of the 10-week programs carried out in the nursing homes involved. To clarify the musical contents and arrangements, each week the workshop leader provided students via email a sheet outlining the schedule of the next session along with the scores and further material (such as lyrics or mp3 files) related to the works to be performed. Before each session a meeting lasting 1 h was scheduled to rehearse the repertoire and to define the roles within the music team. Moreover, a debriefing lasting 30 min was planned at the end of each musical session to analyze the work just done and to discuss the contents of the next session. In this way, the workshop leader and the students developed a repertoire for each nursing home considering the preferences and the wishes expressed by the residents throughout the program.

Depending on the pieces and the musical activities planned by the workshop leader, each student was asked:

• To play his or her own instrument
  ○ making music *for* residents, namely providing short live performances for residents;
  ○ making music *with* residents, namely learning to interact musically with the workshop leader and to adapt tempi and phrasing to the playing of the ensemble of residents.
• To sing or drum along with residents, namely (1) learning the form and arrangement of each work included in the repertoire and (2) learning to interact verbally and non-verbally with residents in appropriate ways.
• To conduct small groups of residents, namely learning to conduct a section of the whole ensemble, communicating with residents and peers and giving effective instructions and feedback.

In cases where iconic symphonic pieces were performed (e.g., Mussorgsky/Ravel: *Pictures at an Exhibition*, Tchaikovsky: *Dance of the Sugar Plum Fairy* from *The Nutcracker*) students and residents played with pre-recorded backing tracks, led by the workshop leader. Finally, students were expected to distribute the percussion instruments according to the required arrangement.

During each musical session, the workshop leader made clear to the residents the objectives of each activity and offered constructive feedback. She discussed with the students the importance of these aspects in the preparation and debriefing stages of each session, in order to make them aware of her techniques and enable them to interact effectively with the residents. In terms of flexibility and adaptability in a complex environment, she highlighted throughout the program the importance of planning activities accurately while being prepared for possible changes of schedule due to unforeseen reactions from the residents. Moreover, students were supported by the teaching team and the carers in dealing with logistical aspects that may affect the musical activities. Examples of this are collaborating to facilitate the displacement of mobility-impaired residents before and after sessions and preparing the room efficiently and without interfering with other activities and people in the facility. In each session, at least one member of the health faculty of the university involved in A4A was present who, in turn, offered support and feedback to the students.

Overall, based on previous literature (Higgins, 2008; Renshaw, 2010; Ruud, 2012; Hallam et al., 2015), the workshop leader aimed to cultivate students’ musical versatility and flexibility, engaging them in a wide repertoire and conveying basic competences in singing, playing percussion instruments and conducting. Moreover, the combined work of the teaching team aimed to prepare students to deal with complex health and living environments, to feed participants’ interests and motivations (Duay and Bryan, 2008), promoting a strong sense of ownership through meaningful communication and attention toward individuals’ backgrounds and expectations (Formosa, 2002; Renshaw, 2010; Hallam et al., 2015).

### 3.2. Characteristics and objectives of the music program

We carried out a 10-week program of group music making in each participating nursing home. The musical activities focused on singing and on drumming using small percussion instruments. Each session lasted 45 min and was opened and closed by greeting ritual songs and a short, live performance provided by students and lasting overall about 15 min. The sessions were held in a space able to accommodate those residents who were willing to participate and that was sufficiently insulated from both other common areas and residents’ private rooms. This, together with the moderate loudness of the instruments we used, made it possible to carry out the sessions without disturbing either the other residents or the facilities staff.

The overriding goal was to make the participants’ experience as enjoyable and engaging as possible. For this reason, the repertoire performed in each nursing home was negotiated with the residents during the sessions. This meant that both the choice of songs and arrangements, as well as the time to be devoted to each activity, were partly defined session by session on the basis of the reactions and proposals of the residents. Moreover, to promote their engagement in each session and throughout the 10-week program, we paid particular attention to the points reported in Table 3.

**TABLE 3:** Key points and related strategies to promote residents’ engagement.

Key points	Strategies
Optimal balance between novelty and routine/confirmatory elements	*Each session included* • Some new elements (i.e., a new piece or arrangement) • Slots to consolidate previous songs • Slots to re-play songs on request
Optimal balance between physically or cognitively demanding tasks and active rest or easy tasks	*Each session included slots where* • Residents listened to students performing the next pieces to play together • The whole group performed simple exercises • Anecdotes about music were told
Development of a repertoire balancing familiar and unfamiliar pieces	*Throughout the program particular care was taken* • To identify and adapt extremely well-known songs • To include songs requested by the participants • To offer to residents the opportunity to approach unfamiliar genres and composers

The repertoire performed in each nursing home ranged widely from classical music to pop songs, folk songs, jazz standards, and world music. For each nursing home, the song list included lyrics in different languages (Italian, German, Spanish, and local Italian dialect) selected on the basis of the different areas of origin of the residents. Moreover, to improve the sense of ownership for participants, they were occasionally invited to act as “teachers”, instructing the students and the workshop leader about the pronunciation of the local Italian dialect or about the form of local songs. In some cases, a particular rhythmic or melodic pattern suggested by a resident was accepted by the group and elaborated collectively.

## 4. Results to date and avenues for development

The seminar reported in this article was designed in the context of a research project conducted over two academic years. Conducting preliminary research was considered an essential step in establishing a training offer that could prepare music university students to act as music facilitators for the older population. A4A specifically considered the population of nursing home residents and engagement with music through group music making. The results of the research are encouraging as they highlighted that music is an important resource for residents and that residents and students perceived mutual benefits by making music together (Paolantonio et al., 2020, 2021, 2022).

This section discusses the next stepsr to be taken to improve the seminar and the overall learning experience of the students. The aspects we consider central to all subsequent initiatives of this type are the tailoring of the training in terms of content for the communities involved, the articulation of learning outcomes and the implementation of a comprehensive process evaluation. With regards to the first of these, we consider crucial to focus on areas related to students’ musical skills and their potential in terms of leadership and co-creation.

In the context of A4A, we did not initially plan to assign students the role of workshop leader. The training focused mainly on preparing them to support the workshop leader and facilitate the involvement of the residents by playing their instruments, singing, drumming, or conducting small groups of residents. We also paid special attention to preparing them to relate properly with a fragile population such as nursing home residents. Nevertheless, on many occasions throughout the program, it was interesting, appropriate or necessary to assign to one student the role of workshop leader, namely conducting the whole group in a specific piece and offering suggestions and feedback. This happened occasionally, especially in the last sessions of the program offered in each nursing home. Moreover, we observed a considerable interest and motivation of the students to be involved as a leader. This highlights the need to redesign the seminar so that each student has his or her own opportunities to conduct. These opportunities should be planned in a systematic and equitable manner. The debriefing scheduled at the end of each session could be supplemented by giving each student feedback on his or her performance as a leader and pointers on what, if anything, to change when they take on this role in subsequent sessions.

The students’ engagement could become more structured in terms of planning the repertoire and the activities for each session. During each rehearsal and debriefing session, the workshop leader took into account the ideas and suggestions of the students who made their proposals on the basis of their observations of residents’ reactions or reflections on the work done. Even so, contributions were occasional and non-systematic. Including regular and explicit assignments focusing on how to organize the next session could empower students in devising content and in observing residents’ reactions and preferences more actively, making their learning experience richer.

A further aspect that can be developed concerns the short live performances offered to residents at the opening of sessions or as moments of active rest. They were appreciated immensely (Paolantonio et al., 2020) and inspired *Music and Words*, a further program engaging both residents and music students (National Activity Providers Association, 2021). In A4A these performances were not linked to each other throughout the program. Planning this content in such a way that students are systematically involved in the definition of the listening program and in a short presentation of it could encourage students to make relevant proposals, proactively organizing themselves to create particular arrangements and acquire basic public speaking skills. All this would increase the artistic value of the program, benefiting the residents but also the students’ musical education.

A4A aimed to explore how residents and music students experience their participation in a community-based program and what effects they perceive on their health, wellbeing and career preparation. The training we offered in A4A was a starting point for designing a seminar to be implemented on a permanent basis. During the first two editions, students were evaluated through a pass/fail grading system based on workshop leader observations and on attendance for at least eight sessions. We collected and analyzed the semi-structured interviews and the oral diaries also with the aim of identifying appropriate learning outcomes and assessment procedures. Table 4 reports the learning outcomes and modalities of evaluation for the next editions of the seminar, defined on the basis of the results of A4A research (Paolantonio et al., 2020, 2022) and the additions just discussed.

**TABLE 4:** Learning outcomes and modalities of evaluation to be implemented in the next editions of the seminar.

Competences the student is expected to have acquired at the completion of the seminar	Evaluation strategies
*Competences for group music making in nursing homes* • Basic skills to play a set of percussion instruments appropriate for residents, to sing and to conduct • Ability to interact musically with the workshop leader • Ability to collaborate effectively and proactively within a music team • Ability to interact with residents • Ability to select and arrange songs and purely instrumental pieces appropriate to engage residents in group music making • Ability to design a 10-week music program	• Observation of the confidence and versatility displayed by the student during the rehearsal and the sessions • Observation of the ability to follow tempo and phrasing cues during rehearsals and sessions • Observation of behavior during the rehearsals and the sessions • Observation of behavior during the sessions • Observation of the quality and relevance of the proposals • Essay
*Competences related to aging processes* Basic knowledge and understanding of concepts related to: • Third and fourth ages • Ageism • Resources of older population	• Essay

The workshop leader will verify the acquisition of competences and regularly consult a member of the nursing home staff to assess students’ ability to properly interact with the residents. On the basis of an essay, the students’ competences in terms of reflexivity, designing musical programs and planning activities will be reviewed by a panel consisting of two teachers and an external expert (e.g., another teacher from the music or health faculty of the university, or a member of the institution hosting the program). This essay will include a hypothetical, revised 10-week program clarifying the objectives, contents and a rationale. We envisage 3 h of individual tutoring and, on the basis of the hours required for the written component, awarding 3 ECTS to students who complete the next iterations of the seminar.

## 5. Avenues for further research

This Curriculum, Instruction, and Pedagogy article has described a seminar aimed at preparing music university students to act as facilitators in nursing homes. We discussed the relevance of engaging in this type of training and offered information for replicating our seminar in other higher music education institutions. Due to the complexity of this field, however, it is important to pursue further research to promote the implementation of this type of training in music universities and to offer a sustainable contribution to the health and wellbeing of residents in nursing homes.

We consider it a priority to develop a curriculum able to offer students interested in this type of work sufficient preparation to act independently as music facilitators for the older population. This idea is also supported by recent studies that emphasize the need to find effective ways to promote the health and wellbeing of residents in nursing homes by facilitating the engagement with music (Garrido et al., 2020; Koivisto and Laes, 2022). This implies a high level in a wide range of musical skills, so that they can sing, drum, arrange, and conduct. Furthermore, considering the heterogeneity of the elderly population (Nelson and Dannefer, 1992; Stone et al., 2017), it is necessary to prepare students to create effective programs outside of nursing homes, addressing for example people in their third age. It is also essential for them to experience a wide range of different kinds of engagement with music, including listening to live music in social settings (Costa and Ockelford, 2019), singing (Lamont et al., 2017; Southcott and Nethsinghe, 2019), group music making (Varvarigou et al., 2013), and using technology (MacRitchie et al., 2021; Taylor et al., 2021). Finally, we consider it crucial to offer students competences in autonomously designing and planning musical programs and in effectively collaborating with all the actors involved in the health and wellbeing of older adults.

Such a perspective requires careful thought with regard to the profiles of the teachers to be involved, the workload for the students and the assessment methods. To deal with this complexity in an effective and sustainable manner, a promising approach is the creation of a teaching module in the context of continuing education.^2^ A proper subdivision of subjects, learning outcomes and workload could make it possible for graduate students to specialize in this area, while Bachelor’s and Master’s degree students could approach it by enrolling in an elective seminar such as that described above. We designed it to include a small amount of preparatory training compared with other programs (David et al., 2018; Smilde et al., 2019) aiming to encourage these students to explore the potential of community-based programs and the benefits that engaging with music can provide to the older population.

Implementing this type of training steadily and beyond the geographical area where A4A was carried out could offer significant benefits to students, music universities and local communities, promoting the development of “innovative ways of supporting the holistic wellbeing of all participants, integrating the whole professional community into the ongoing creation of novel musical spaces” (Koivisto and Laes, 2022). As for students, involvement in community-based programs can foster transformative learning (Perkins et al., 2015) and meaningful learning experiences in terms of musical and extra-musical skills (Renshaw, 2010; Smilde, 2018), with positive effects on employability, identity, and health and wellbeing (Triantafyllaki and Anagnostopoulou, 2013; Smilde et al., 2019; Paolantonio et al., 2022). Music universities could strengthen their role and visibility at a local level, create partnerships with stakeholders and initiate or increase collaborations with other university departments. Crucially, on the basis of the rich literature demonstrating the benefits of music on the health and wellbeing of older adults (Creech et al., 2014; Fancourt and Finn, 2019), it can be expected that the development of music activities could improve the health and wellbeing of older adults. This picture is in line with the concept of “Musicians as makers in society” promoted by the Association Européenne des Conservatoires (Gaunt et al., 2021), which emphasizes the need for a “paradigm shift at institutional, curriculum and pedagogical levels” (p.16) and the urgency of new frameworks able to strengthen the role of the next generations of professional musicians and higher music education institutions in society.

The complexity of this field in pedagogical, organizational and artistic terms makes it necessary to do further research. Among the many questions still open, we consider two avenues in particular. One concerns the impact of musical activities on the formal and informal careers of residents. Crawford et al. (2013) proposed the concept of *creative practice as mutual recovery* arguing that the implementation of artistic programs in care settings can generate wellbeing for all involved. This idea seems to be confirmed in the case of music programs carried out in hospital wards (Smilde et al., 2019) and in mental health settings (Perkins et al., 2016; Callahan et al., 2017). Mutual benefits have been observed among residents and students who took part in A4A (Paolantonio et al., 2020, 2022), but the kinds and the extent of potential benefits of music interventions on residents’ relatives, nurses, and volunteers active in nursing homes are still be clarified. The second avenue to investigate refers to the use of digital platforms, which have recently begun to be explored in music programs addressing residents in nursing home (Taylor et al., 2021) and community dwellings (MacRitchie et al., 2021). The potential of these approaches requires further investigation, particularly in terms of the devices and platforms to be used, their salience with older users and optimal usage times and durations. In light of the difficulties experienced during the COVID-19 pandemic also in terms of isolation and loneliness, and considering the growing number of people over 65 accustomed to using computers and smartphones for learning and joining communities, doing research in this direction appears to be crucial also for music universities.

Considering the work reported in this article, training musicians to work in community-based settings, specifically those for older adults, represents a sound educational investment by higher music education institutions. Music students are able to achieve competences relevant for professional, portfolio careers while offering local older populations music programs relevant to their health and wellbeing, fostering their own wellbeing in the process. In this way, exciting new educational, artistic and health-related opportunities and partnerships can be forged, for the benefit of individuals, communities and society as a whole.

## Data availability statement

The original contributions presented in this study are included in the article/supplementary material. Further inquiries can be directed to the corresponding author.

## Author contributions

All authors listed have made a substantial, direct, and intellectual contribution to the work, and approved it for publication.

## Footnotes

1 Here, we use “seminar” to refer to curriculum-based elective training activities of varying duration that are offered to Bachelor’s and Master’s students to complete their education and to reach the required number of credits for their degrees. Depending on the international context, the term is comparable to “module” or “course”—that is, a component of a larger program of study. The institution described in this article implements the European Credit Transfer and Accumulation System (ECTS). A Bachelor’s degree corresponds to 180 ECTS and a Master’s degree to 120 ECTS.

2 Here, we refer to “continuing education” as post-graduate training enabling specialization in particular professional areas.
